# Prenatal diagnosis of megaduodenum using ultrasound: a case report

**DOI:** 10.1186/s12884-021-03843-0

**Published:** 2021-05-11

**Authors:** Kaihui Zeng, Dongmei Li, Yao Zhang, Chengcheng Cao, Ruobing Bai, Zeyu Yang, Lizhu Chen

**Affiliations:** 1 Department of Ultrasound, Shengjing Hospital of China Medical University, No.36 Sanhao St, Shenyang, 110004 Liaoning China; 2 Department of Ultrasound, Shenyang Women’s and Children’s Hospital, Shenyang, Liaoning China; 3 Department of Pathology, Shengjing Hospital of China Medical University, Shenyang, Liaoning China; 4 Department of Radiology, Shengjing Hospital of China Medical University, Shenyang, Liaoning China

**Keywords:** Megaduodenum, Fetus, Prenatal diagnosis, Ultrasound

## Abstract

**Background:**

Congenital megaduodenum is a rare disorder; however, its prenatal diagnosis has not been reported previously. We report the case of an abdominal cystic mass in a fetus that was later diagnosed as megaduodenum.

**Case presentation:**

An abdominal cystic mass was found during ultrasonography of a fetus at 11 weeks of gestation. The mass progressively enlarged with gestation. The amniotic fluid volume decreased and then returned to normal. During the last prenatal ultrasound examination, the mass was observed communicating with the stomach; therefore, duodenal dilation was suspected. Finally, the patient was diagnosed with megaduodenum caused by a developmental defect in the nerve plexus.

**Conclusions:**

Congenital megaduodenum is a differential diagnosis of massive fetal abdominal cystic masses. Ultrasound examinations of such masses communicating with the stomach may help determine the diagnosis.

**Supplementary Information:**

The online version contains supplementary material available at 10.1186/s12884-021-03843-0.

## Background

Megaduodenum is a rare clinical syndrome caused by mechanical or functional abnormalities, which is characterized by remarkable expansion of the duodenum. Usually, there is significant expansion of the descending part, horizontal part, and ascending part of the duodenum, and the diagnosis can be made when the diameter of the duodenum is larger than 5 cm [1]. Megaduodenum was first reported by Melchior [2] in 1924; however, only a few such cases in adults and children have been reported [3–7]. The prenatal diagnosis of megaduodenum has not been previously reported. We report the case of an abdominal cystic mass in a fetus detected at 11 4/7 weeks of gestation that was later diagnosed as megaduodenum following postnatal surgery and histopathological examination.

## Case presentation

**Fig. 1:**
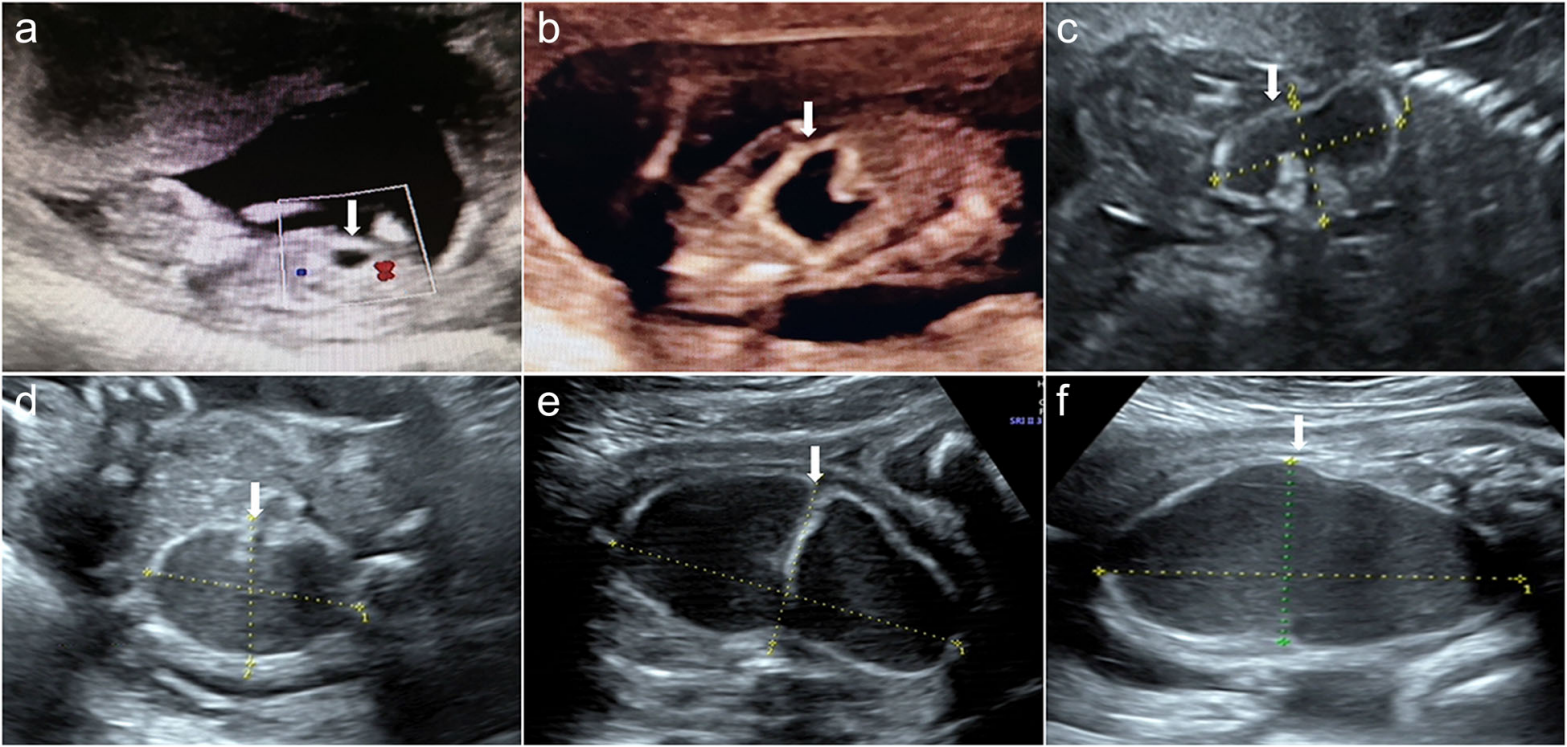
Ultrasound findings of six prenatal examinations: **a** 11 4/7 weeks of gestation; **b** 15 4/7 weeks of gestation; **c** 20 3/7 weeks of gestation; **d** 25 3/7 weeks of gestation; **e** 29 3/7 weeks of gestation; **f** 33 3/7 weeks of gestation. The arrow indicates the cystic mass


A 30-year-old woman (gravida 4, para 0) who conceived by in vitro fertilization presented with a fetus with a cystic abdominal mass detected on fetal nuchal translucency examination at 11 4/7 weeks of gestation. She was referred to our hospital for a fetal ultrasound consultation at 20 3/7 weeks of gestation. The findings of the ultrasound examination are shown in Table 1 and Fig. 1. The cystic mass enlarged with gestational age. During the last prenatal examination at 33 3/7 weeks of gestation, emptying of the stomach was observed. Fluid passed through the pylorus into the cystic mass; the fluid content in the mass was thick, allowing clear visualization of the fluid flow from the stomach into the cystic mass (Additional file 1). Based on this finding, duodenal dilation was suspected. Amniocentesis was performed, and the karyotype was found to be normal. Comparative genomic hybridization (CGH) -array revealed microdeletions on chromosome 16 [del (16) (p12.2) chr16: g. 22000000_22440000 del] and microduplications on chromosome 9 [dup (9) (p24.1) chr9: g. 6320000_7420000 dup]. Public databases of DGV, DECIPHER, OMIM, ClinGen, UCSC, gnomAD, and PubMed were checked. The microdeletions on chromosome 16 were at high risk of pathogenic variations that could manifest as growth restriction, language delay, mild to moderate mental deficiency, mental and behavioral abnormalities, congenital heart disease, and bone malformations; however, their associations with megaduodenum have not been reported. Microduplications on chromosome 9 were regarded as variants of unknown significance. Because the abnormalities on chromosome 16 were inherited from the father who presented with a normal phenotype, the woman decided to continue the pregnancy.

**Table 1:** Prenatal ultrasound findings of the fetus

Gestational age	11 4/7 weeks	15 4/7 weeks	20 3/7 weeks	25 3/7 weeks	29 3/7 weeks	33 3/7 weeks
Gestational age estimated by ultrasound	11 5/7 weeks	15 1/7 weeks	19 3/7 weeks	23 3/7 weeks	28 2/7 weeks	33 4/7 weeks
Cyst size, cm	0.7 × 0.6 × 0.5	1.5 × 1.0 × 0.7	3.3 × 1.7 × 1.6	4.6 × 4.8 × 2.5	7.1 × 6.7 × 4.0	9.0 × 7.9 × 5.8
Internal echo findings of the cyst	Oval shape, anechoic	Irregular shape, anechoic	Irregular shape, homogeneous low-level echogenicity of the cyst fluid, echogenic wall	Irregular shape, homogeneous low-level echogenicity of the cyst fluid, echogenic wall	Irregular shape, homogeneous low-level echogenicity of the cyst fluid, echogenic wall	Irregular shape, homogeneous low-level echogenicity of the cyst fluid, echogenic wall
Amniotic fluid volume, DVP	3.6 cm	3.4 cm	3.2 cm	2.5 cm	2.7 cm (AFI, 7)	4.7 cm (AFI, 13)
Additional findings	–	–	–	–	–	The cystic mass communicated with the stomach and the cervix was open

*AFI* amniotic fluid index, *DVP* depth of the vertical pocket

**Additional file 1.** Gastric contents emptying into the cystic mass. We clearly observed the emptying of the stomach and fluid passing through the pylorus into the cystic mass.

**Fig. 2:**
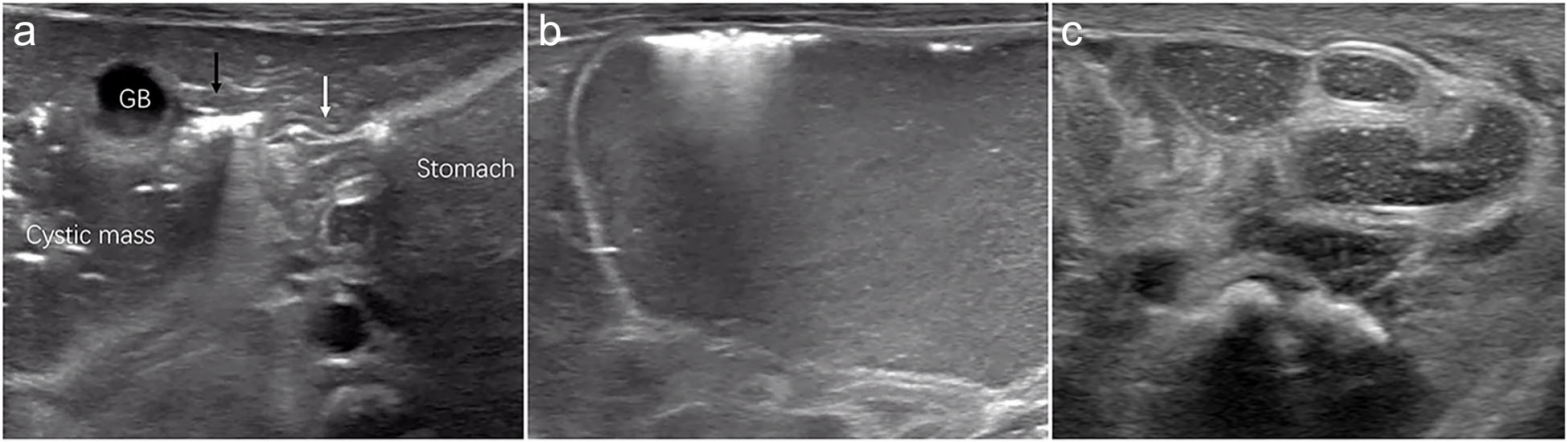
Ultrasound images of the child at 10 h after birth. **a** The cystic mass is connected to the stomach through the pylorus (*white arrow*) and upper duodenum (*black arrow*). The cystic mass and gas-like echoes are seen in the upper duodenum. **b** A giant cystic mass, approximately 7.3 × 5.1 × 3.9 cm in size, is located in the right abdominal cavity and has ground-glass echogenicity. Gas-like echoes also appear in the cystic mass. **c** The colonic bowel is compressed to the left side of the abdomen. No obvious abnormalities in the colon and rectum are seen

**Fig. 3:**
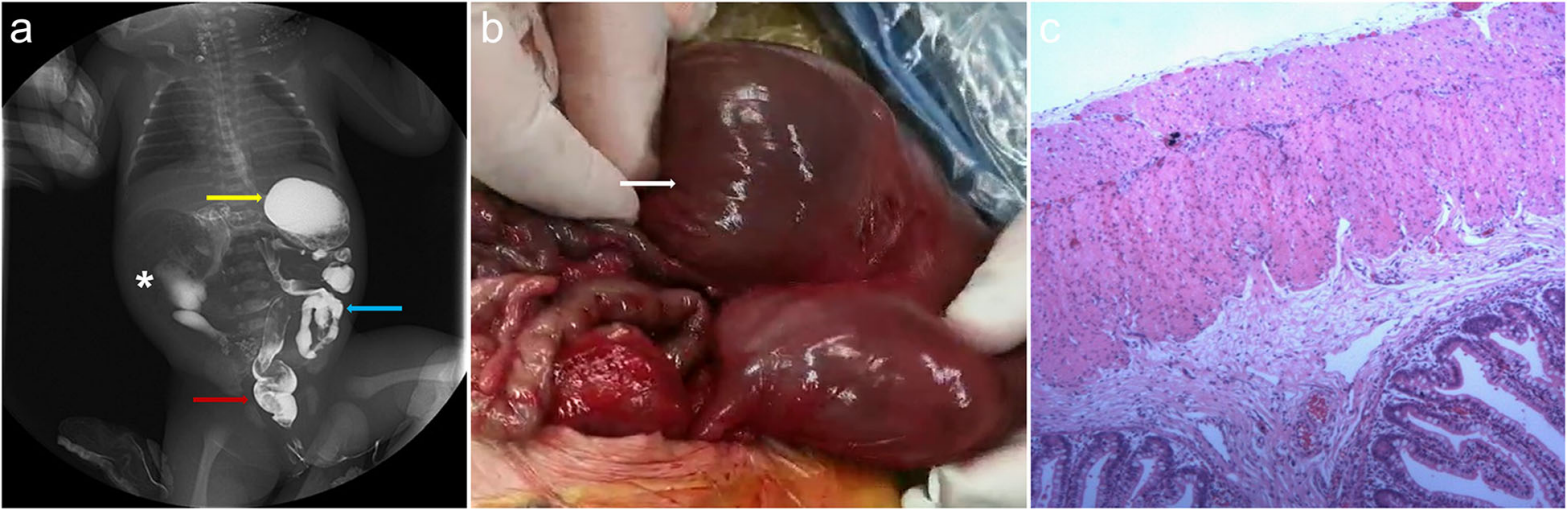
**a** Images of the upper gastrointestinal contrast, a giant cystic mass containing gas (*asterisk*), a normal stomach (*yellow arrow*), colon (*blue arrow*), and rectum (*red arrow*). **b** Intraoperative findings of a giant duodenal loop (*white arrow*). **c** Histopathological image of megaduodenum (hematoxylin and eosin stain, × 40)

Spontaneous preterm labor occurred at 33 3/7 weeks of gestation. Because of the breech position of the fetus, cesarean delivery of a female neonate (birth weight, 2240 g) was performed at 34 1/7 weeks of gestation. The child was kept on fasting, and transabdominal ultrasonography was performed 10 h after birth. The ultrasound findings are presented in Fig. 2. Upper gastrointestinal contrast showed that the gastric antrum had moved upward, the descending part of the duodenum was slightly compressed, the duodenum had expanded, and the contrast agent passed through the duodenum smoothly (Fig. 3a).

The child underwent surgery 2 days after birth. Exploratory laparotomy revealed a giant duodenal loop with a diameter of approximately 6 cm and intestinal malrotation (Fig. 3b). The proximal jejunum had a diameter of approximately 0.5 cm, and no obvious obstruction was observed. The large duodenal loop was surgically removed, and duodenojejunostomy was performed. Most of the intermuscular plexus was not visible and ganglion cells were not observed on histopathological examination (Fig. 3c).

The child was in a good condition and was discharged from the hospital on day 13 after surgery. During the follow-up examination at 3 months after surgery, the child was found to be in good health.

## Discussion and conclusions

Megaduodenum is a clinical syndrome with multiple causes; the condition is divided into two categories, namely, obstructive and non-obstructive, based on whether there is genuine obstruction at the distal end of the duodenum. Obstructive factors may be extra-duodenal (superior mesenteric artery syndrome, annular pancreas, tumors, congenital bands), related to the duodenal wall (duodenal diverticulum, duodenum inflammation), or related to the duodenal lumen (atresia, gallstones, bezoars, duodenal septum stenosis, parasite infections) [8]. Non-obstructive factors include congenital diseases (familial inherited megaduodenum, congenital ganglion cell deficiency) and acquired diseases (systemic lupus erythematosus, amyloidosis, diabetes) that affect the smooth muscles of the intestine or the plexus of the intestinal wall [9, 10]. This case was attributed to ganglion dysplasia, which is a non-obstructive factor.

To the best of our knowledge, this is the first report of a case of fetal megaduodenum. The dilated intestine lost its normal shape and underwent massive expansion during the second and third trimesters. The expanded intestine was connected to the stomach, which was not dilated and could be emptied. In addition, the colon and rectum were found to be normal, and polyhydramnios did not occur. Because this case was caused by abnormal development of the nerve plexus, its indications appeared early on ultrasound. Furthermore, the lower duodenal obstruction was not obvious; therefore, the manifestations found during ultrasound were different from those of obstructive diseases. Fetal megaduodenum must be distinguished from meconium pseudocyst and duodenal stenosis or atresia; the main identification points are summarized in Table 2.

**Table 2:** Differential diagnosis of megaduodenum

Differential diagnosis	Time of discovery	Stomach size	Communication between the mass and stomach	Amniotic fluid	Mass characteristics	Other manifestations on ultrasound
Megaduodenum	Can be found during early pregnancy	Normal	Yes	Normal	Enlargement with gestational week, irregular shape, echogenic wall	–
Duodenal stenosis or atresia [11, 12]	Usually found during the second trimester	Dilated	Yes	Often accompanied by polyhydramnios	Spherically dilated duodenum	“Double-bubble” sign, a shriveled bowel can sometimes be observed at the distal end
Meconium pseudocyst [13, 14]	Mid-to-late pregnancy	Normal	No	Often accompanied by polyhydramnios	Large cystic mass with echogenic walls and some septa	Ascites, scattered intra-abdominal calcifications

Patients with megaduodenum often present with recurrent abdominal distention, nausea, vomiting, diarrhea, malnutrition, and dystrophia [15]. Because this case was diagnosed before birth, the child was kept fasting immediately after birth and she had no other clinical manifestations except abdominal distension. The earliest reported case of megaduodenum was discovered in a 3-month-old infant; the clinical symptoms included failure to gain weight, an abdominal mass, nausea, and vomiting [5]. That child was followed up for 3 years after surgery, gained adequate weight, and was able to lead a healthy life [5].

Megaduodenum is mainly treated by surgery. The tension-free duodenal wall should be completely removed to prevent postoperative functional anastomotic obstruction. For megaduodenum secondary to other diseases, the primary disease must be addressed first [6, 8, 16]. In our case, extreme dilation of the duodenum was caused by a defect in the development of the nerve plexus, and radical duodenectomy was performed. Three months after surgery, the child was in good health. Further follow-up is required to determine the long-term prognosis.

In conclusion, although congenital megaduodenum is rare, it should be considered as part of the differential diagnoses of fetal abdominal cystic masses. In particular, if a giant cystic mass communicating with the stomach is detected on ultrasonography, the prenatal diagnosis of congenital megaduodenum should be considered.

## Data Availability

All data analyzed during this study are included in this report.

## Abbreviations

AFI: Amniotic fluid index
CM: Cystic mass
DVP: Depth of the vertical pocket
GB: Gallbladder
ST: Stomach
